# G_s_ G Protein–Coupled Receptor Signaling in Osteoblasts Elicits Age-Dependent Effects on Bone Formation

**DOI:** 10.1002/jbmr.3

**Published:** 2010-01-08

**Authors:** Edward C Hsiao, Benjamin M Boudignon, Bernard P Halloran, Robert A Nissenson, Bruce R Conklin

**Affiliations:** 1 Gladstone Institute of Cardiovascular DiseaseSan Francisco, CA, USA; 2 Department of Medicine, University of CaliforniaSan Francisco, CA, USA; 3 Endocrine Research Unit, VA Medical Center and Departments of Medicine and Physiology, University of CaliforniaSan Francisco, CA, USA; 4 Department of Cellular and Molecular Pharmacology, University of CaliforniaSan Francisco, CA, USA

**Keywords:** Fibrous Dysplasia, Rassl, G Protein–coupled Receptor, G_S_ Signaling, Age-dependent G_S_ Activation, Osteoblasts, Inbred Mice

## Abstract

Age-dependent changes in skeletal growth are important for regulating skeletal expansion and determining peak bone mass. However, how G protein–coupled receptors (GPCRs) regulate these changes is poorly understood. Previously, we described a mouse model expressing Rs1, an engineered receptor with high basal G_s_ activity. Rs1 expression in osteoblasts induced a dramatic age-dependent increase in trabecular bone with features resembling fibrous dysplasia. To further investigate how activation of the G_s_-GPCR pathway affects bone formation at different ages, we used the tetracycline-inducible system in the *ColI(2.3)*^+^/*Rs1*^+^ mouse model to control the timing of Rs1 expression. We found that the Rs1 phenotype developed rapidly between postnatal days 4 and 6, that delayed Rs1 expression resulted in attenuation of the Rs1 phenotype, and that the Rs1-induced bone growth and deformities were markedly reversed when Rs1 expression was suppressed in adult mice. These findings suggest a distinct window of increased osteoblast responsiveness to G_s_ signaling during the early postnatal period. In addition, adult bones encode information about their normal shape and structure independently from mechanisms regulating bone expansion. Finally, our model provides a powerful tool for investigating the effects of continuous G_s_-GPCR signaling on dynamic bone growth and remodeling. © 2010 American Society for Bone and Mineral Research.

## Introduction

Osteoporosis affects over 10 million people in the United States and contributes to 1.5 million fractures each year.(1) A crucial determinant of osteoporosis and fracture risk in adults is thought to be the acquisition of peak bone mass during childhood and puberty.(2–4) Osteoblasts and their precursors play key roles in regulating bone development and acquisition of peak bone mass.(5) Although certain G protein–coupled receptors (GPCRs) expressed in osteoblasts [e.g., parathyroid hormone receptor (PTHR1)(6) and HTR1b serotonin receptor(7)] are involved in regulating bone mass, the exact in vivo roles of GPCR signaling during skeletal growth have not been clearly elucidated.

GPCRs signal through a select number of pathways, including the G_s_ and G_i_ pathways that influence intracellular cyclic AMP (cAMP) levels.(8) We described a synthetic biology approach(9) for activating the G_s_ pathway that uses the strong basal G_s_ signaling activity of Rs1, an engineered receptor activated solely by a synthetic ligand (RASSL).(10,11) RASSLs are engineered receptors that no longer respond to endogenous hormones but can be activated by synthetic small-molecule drugs. They have proven useful for studying the roles of G protein signaling in complex systems.(12–15)

We found that when Rs1 was expressed continuously in mouse osteoblasts from gestation until adulthood, skeletal bone mass and trabecular bone formation were dramatically greater than in wild-type mice.(11) Cortical bone was lost, and serum markers of bone turnover were greatly increased. In addition, the normal bone marrow space was obliterated and replaced with small uniform cells morphologically resembling young osteoblasts. In contrast, when Rs1 was expressed beginning at 4 weeks of age, no grossly abnormal skeletal phenotype was evident through 30 weeks of age. These findings suggested that receptor-mediated G_s_ signals in osteoblasts have age-dependent effects on bone formation. In addition, evidence from human diseases and animal models suggest that osteoblast function is markedly different in children than in adults.(16–21)

**Fig. 1:**
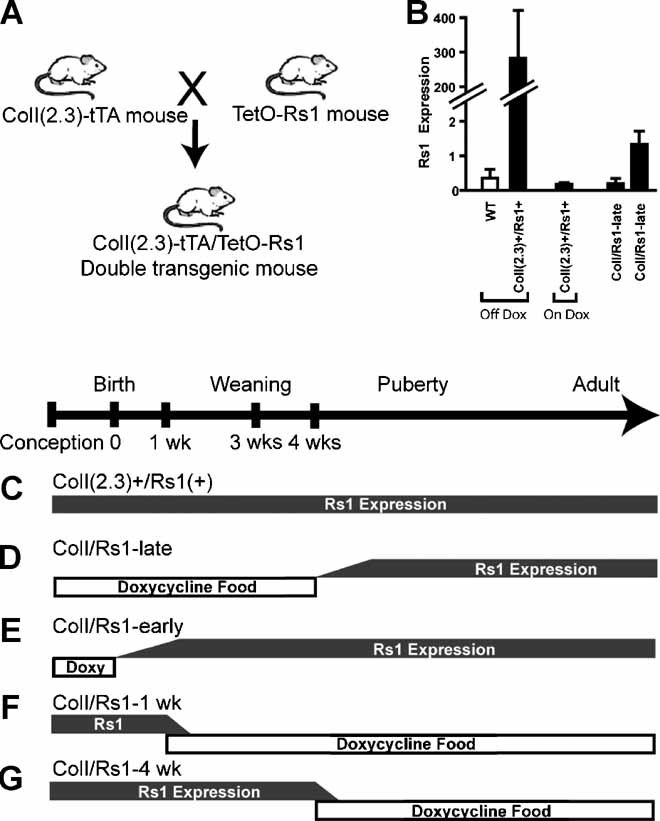
Timing of Rs1 expression in the *ColI(2.3)-tTA*/*TetO-Rs1* mice. (*A*) We used a doxycycline-regulated expression system to focus on the effects of basal G_s_ signaling induced by Rs1 expression without ligand activation. (*B*) Rs1 expression was allowed by feeding the mice regular doxycycline-free food. Six-week-old *ColI(2.3)*^+^/*Rs1*^+^ mice raised on regular food (Off Dox) had high levels of Rs1 expression. We suppressed Rs1 expression by giving doxycycline in the mouse chow (On Dox). Mice born and raised on doxycycline-containing food and subsequently switched to doxycycline-free food at 4 weeks of age show low but variable levels of Rs1 expression at 6 weeks of age (*ColI/Rs1*-late). Expression levels from femora of representative mice are shown. Portions of this figure use RNA samples from mice used in Fig. S5 as published.(11) Values are mean ± 1 SD of technical triplicates. (*C–G*) The pattern of doxycycline food administration and Rs1 expression for each experimental manipulation is indicated in gray. (*C*) *ColI(2.3)*^+^/*Rs1*^+^ mice: Rs1 expression was allowed from gestation through adulthood by feeding the pregnant mothers and their pups regular chow. (*D*) *ColI/Rs1*-late mice: Rs1 expression was suppressed through the first 4 weeks of development by feeding doxycycline chow to mothers during mating and to their pups after weaning. We switched the mice to regular chow at 4 weeks of age. (*E*) *ColI/Rs1-*early mice: Rs1 expression was allowed after birth by feeding the mother doxycycline chow from mating until delivery. The nursing mothers were switched to regular chow at delivery. (*F*) *ColI/Rs1*–1 week mice: Rs1 expression was allowed until 1 week of age by feeding the mothers regular chow from mating until 1 week after birth. The nursing mothers were switched to doxycycline chow to suppress Rs1 expression in the nursing pups. Weaned animals were maintained on doxycycline chow. (*G*) *ColI/Rs1*–4 week mice: Rs1 expression was allowed from gestation through 4 weeks of age by feeding mothers regular chow from mating through weaning. The pups were given regular chow until 4 weeks of age. Rs1 expression then was suppressed by switching the pups to doxycycline chow.

In this study, we asked how the timing of G_s_ signaling affects the formation of skeletal bone at different ages. We used Rs1 to activate the G_s_ signaling pathway in osteoblasts in mice at different ages in four experiments (Fig. 1). First, we identified a window of osteoblast responsiveness to Rs1 expression by examining when the Rs1-induced trabecular bone phenotype begins. Second, we narrowed the window of Rs1 expression necessary for developing the trabecular overgrowth phenotype by delaying Rs1 expression until after birth. Third, we assessed whether expression of Rs1 after 4 weeks of age could lead to long-term attenuation of the Rs1 phenotype in adult mice. Finally, we determined whether the Rs1-induced bone phenotype could be reversed if Rs1 expression was discontinued after formation of the abnormal trabecular bone in adult mice. Our findings demonstrate the utility of the Rs1 mouse model for controlling the timing and tissue-specific activation of G_s_ signaling as a way to understand the age-dependent effects of continuous G_s_ signaling by GPCRs.

## Methods

### Animal studies

All transgenic mouse studies were approved by and performed in accord with the Institutional Animal Care and Use Committee and the Laboratory Animal Research Center at the University of California, San Francisco. The *ColI(2.3)-tTA*/*TetO-Rs1* double transgenic mice were generated by heterozygote crosses of mice carrying the *TetO-Rs1* transgene with mice carrying the *ColI(2.3)-tTA* transgene as described previously.(11) Transgene expression was suppressed by continuous administration of doxycycline-impregnated mouse chow (DoxDiet 200 mg/kg; BioServ, Frenchtown, NJ, USA) during the times indicated in Fig. 1. Transgene expression was activated by switching the mice to regular mouse chow without doxycycline (LabDiet 5053, PMI Nutrition, St. Louis, MO, USA) at the times indicated in Fig. 1. Full transgene expression was expected to occur within 1.5 to 2 weeks of doxycycline withdrawal.(12,22) Transgene expression was verified by quantitative polymerase chain reaction (qPCR) in representative animals from each cohort (see Figs. 1*B*, 3*C*, 5*C*, and 7*F*). Neonatal pups analyzed in Fig. 3 were not manipulated until the day of tissue collection to minimize any risk of maternal distress or cannibalism. Genotyping and selection of the appropriate *ColI(2.3)*^+^/*Rs1*^+^ and control pups were done after tissue collection, or after 4 weeks of age for adult animals. No alterations in litter size, pup health, or maternal health were observed in our experiments. Both males and females were analyzed together in our experiments because no sex-dependent differences were observed.

**Fig. 2:**
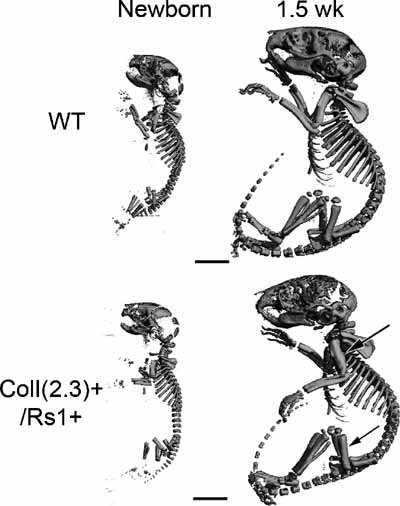
Mice expressing Rs1 from gestation showed no gross skeletal patterning defect. Note the subtle changes in bone size in the 1.5-week-old *ColI(2.3)*^+^/*Rs1*^+^ mice with mild thickening in the diaphyses of the femora and humeri (*arrows*). Scale bar = 5 mm.

**Fig. 3:**
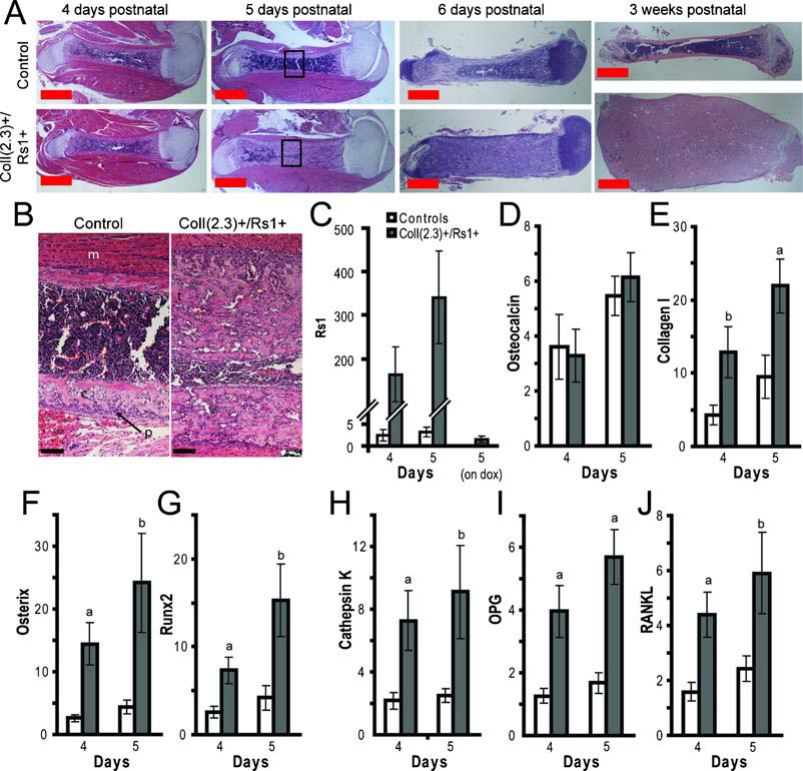
Rs1-induced bone formation occurs between days 4 to 6 in postnatal skeletal growth. (*A*) Representative hematoxylin and eosin histology of paraffin-embedded decalcified femur sections of 4-, 5-, and 6-day-old and 3-week-old *ColI(2.3)*^+^/*Rs1*^+^ or control (wild-type or single-transgenic) mice. Note the increased trabecular bone, decreased cortical bone, and narrowed bone marrow space in the day 5 and 6 femora reminiscent of the changes seen in the 3-week-old mice. Skeletal muscle was left in place for 4- and 5-day-old samples to preserve adjacent bone morphology during processing. p = periosteum; s = bone marrow space; c = cortical bone; t = trabecular bone; m = skeletal muscle. Scale bar = 1 mm. (*B*) High-magnification images of day 5 control and *ColI(2.3)*^+^/*Rs1*^+^ femora (boxed regions in panel *A*) showing the increased trabecular bone resulting from Rs1 expression. (*C–J*) Relative expression levels by quantitative PCR of whole humeri from *ColI(2.3)*^+^/*Rs1*^+^ (*filled bars*) and control littermates (*open bars*). For the quantitative PCR, *n* = 6 control and 6 mutant 4-day-old mice; *n* = 6 control and 5 mutant 5-day-old mice. Expression levels were determined in technical triplicates for each mouse and normalized to GAPDH. Values are mean ± 1 SEM. *a:* *p* < .05 versus controls; *b:* *p* < .1 versus controls. (*C*) Rs1 expression in 4- and 5-day-old *ColI(2.3)*^+^/*Rs1*^+^ pups and littermate controls. Five-day-old *ColI(2.3)*^+^/*Rs1*^+^ pups from mothers fed doxycycline-containing food (day 5, on dox) demonstrate suppression of Rs1 expression, indicating that doxycycline fed to nursing mothers crossed adequately into breast milk. *n* = 4 *ColI(2.3)*^+^/*Rs1*^+^ pups maintained on doxycycline. (*D*, *E*) Osteocalcin and collagen I expression, markers of mature osteoblasts. (*F*, *G*) Osterix and runx2 expression, markers of immature osteoblasts. (*H–J*) Cathepsin K, OPG, and RANKL expression, markers of osteoclastogenesis.

Gene expression analysis was performed on RNA isolated from the right femur or right humerus of adult experimental animals, as indicated in each figure legend. For the experiments in Fig. 3, both humeri from the same 4- or 5-day-old animal were combined to maximize RNA yield. Briefly, whole bones were dissected away from surrounding soft tissue and frozen in liquid nitrogen. Bones for each experiment were batch processed by crushing (multisample Bio-Pulverizer, Research Products International, Mt. Prospect, IL, USA) followed by homogenization (4.5 mm Tissue Tearor, Research Products International) in RNAStat-60 (Iso-Tex Diagnostics, Friendswood, TX, USA). cDNA was generated using the SuperScript III First Strand Synthesis kit (Invitrogen, Carlsbad, CA, USA) as directed by the manufacturer. Expression was assayed using SybrGreen primers as described previously,(11) Sybr green primers for RANKL (forward = TTGCACACCTCACCATCAAT; reverse = TCCGTTGCTTAACGTCATGT) and OPG (forward = CAGAGACCAGGAAATGGTGAA; reverse = AAGCTGCTCTGTGGTGAGGT) or TaqMan primers (Mm99999913_g1 for GAPDH, Mm00484036_m1 for cathepsin K, Mm0081666_g1 for collagen 1a1, Mm00504574_m1 for osterix, and Mm00501578_m1 for runx2). All samples were assayed in technical triplicate, and expression levels were normalized to GAPDH. All qPCR reactions were run on an Applied Biosystems (Foster City, CA, USA) 7900HT real-time thermocycler.

### Bone densitometry and imaging

Mice identified for dual-energy X-ray absorptiometry (DXA) to measure whole-body areal bone mineral density (aBMD) were anesthetized with inhaled isofluorane (1.5% to 2% in oxygen) and scanned on a GE Lunar Piximus2 (Madison, WI, USA) at predetermined times. Mice that underwent whole-mouse or ex vivo femur micro-computed tomographic (µCT) scans were sacrificed before scanning on a Scanco vivaCT-40 µCT scanner, (SCANCO, Waynes, PA, USA). Femora for ex vivo scanning were fixed in 10% neutral buffered formalin (Fisher Scientific, Fairlawn, NJ, USA) for 24 hours and stored in 70% ethanol. Images were obtained at an X-ray energy of 55 kV, with a voxel size of 76 or 10.5 µm and integration times of 200 or 1000 ms for whole-body images or ex vivo femur images, respectively. Because of age-dependent variations in bone mineralization, segmentation values for whole-animal µCT were determined empirically as 0.1/1/100 (gauss sigma/gauss support/threshold in mg HA/ccm) for newborns and 1.5-week-old animals. Segmentation values of 0.7/1/250 were used for the ex vivo femur CT scans.

### Bone histology

Mice identified for histomorphometry were injected with 15 mg/kg calcein (Sigma-Aldrich, St. Louis, MO, USA) 7 days before harvesting and with 90 mg/kg xylenol orange (Sigma-Aldrich) 2 days before harvesting to label areas of active bone mineralization. Harvested bones were processed for von Kossa staining, tartrate-resistant acid phosphatase (TRAP) immunostaining, and fluorescence microscopy as described previously.(11) Bones for hematoxylin and eosin staining were decalcified for 12 to 48 hours in 10% EDTA/PBS before paraffin embedding, sectioning, and staining by the Gladstone Histology Core.

### Serum analysis

Blood was collected from euthanized mice by cardiac puncture and processed in MicroTainer serum separator tubes according to the manufacturer's instructions (BD Biosciences, San Jose, CA, USA). Routine blood analysis was carried out by Antech Diagnostics (Irvine, CA, USA). Serum osteocalcin and pyridinoline measurements were carried out using the mouse osteocalcin EIA Kit BT-470 from BTI (Stoughton, MA, USA) and the MetraBiosystems SerumPYD Kit 8019 (Palo Alto, CA, USA) according to manufacturers' directions.

### Statistical analysis

Two-tailed Student's *t* tests with unequal variances were performed using Microsoft Excel 2003. ANOVA analysis was performed using JMP8 (Version 8.0, SAS Institute, Cary, NC, USA).

## Results

### Rs1-induced bone formation in early postnatal skeletal growth

We previously reported that Rs1-induced basal G_s_ signaling in osteoblasts of *ColI(2.3)*^+^/*Rs1*^+^ mice increased trabecular bone and decreased cortical bone in lesions reminiscent of fibrous dysplasia of the bone.(11) In addition, increases in the whole-body BMD of *ColI(2.3)*^+^/*Rs1*^+^ mice expressing Rs1 were detectable only after 3 weeks of age.(11) To determine if Rs1-induced trabecular bone formation occurs earlier in postnatal growth and if Rs1 affects the patterning of the normal bone structures, we used µCT, histology, and gene expression to analyze bone formation in young mice expressing Rs1 from gestation.

µCT imaging showed no gross differences in skeletal patterning or overall bone shape before 1.5 weeks of age (see Fig. 2). At 1.5 weeks of age, small changes in bone structure were observed, primarily in the long bones of the mice. Since µCT analysis in prepubertal mice is complicated by rapidly changing bone mineral content during postnatal growth, we assessed bone structure by performing histology on femora from *ColI(2.3)*^+^/*Rs1*^+^ mice at 4 to 6 days after birth.

Histology revealed that femora of the *ColI(2.3)*^+^/*Rs1*^+^ mice had normal periosteum, bone collar, and bone marrow cells through 4 days of postnatal growth (see Fig. 3*A*) despite detectable Rs1 expression (see Fig. 3*C*). However, by 5 days of postnatal growth, femora from the *ColI(2.3)*^+^/*Rs1*^+^ mice showed increased trabecular bone, decreased cortical bone, and narrowed bone marrow space (see 3*A, B*). By 6 days, the femora fully recapitulated the trabecular bone replacement of the entire bone as described in 3-week-old *ColI(2.3)*^+^/*Rs1*^+^ mice(11) (see 3*A*).

Gene expression analysis from 4 day old *ColI(2.3)*^+^/*Rs1*^+^ and control mice was used to examine the expression of genes marking mature (osteocalcin and collagen I) and immature osteoblasts (osterix and runx2) (see Fig. 3*D–G*). Osteocalcin levels were unchanged in the 4-day-old *ColI(2.3)*^+^/*Rs1*^+^ mice. However, collagen I, osterix, and runx2 all showed increased levels in the *ColI(2.3)*^+^/*Rs1*^+^ mice despite the lack of an obvious histologic phenotype. By day 5, expression of all these genes was greater in the bones of *ColI(2.3)*^+^/*Rs1*^+^ mice than in those of wild-type mice. In addition, expression levels of cathepsin K (a marker of osteoclasts) as well as osteoprotegerin (OPG) and RANKL (both regulators of osteoclast activity) were increased in the *ColI(2.3)*^+^/*Rs1*^+^ mice at both 4 and 5 days of age. The RANKL/OPG ratios, used as a measure of osteolytic balance,(23) were not significantly different in the *ColI(2.3)*^+^/*Rs1*^+^ mice between days 4 and 5 (1.25 versus 1.11, respectively).

These results show that continuous G_s_ signaling induced by Rs1 in osteoblasts leads to increased trabecular bone formation starting after day 4 of postnatal growth and increased expression of early osteoblast markers. In contrast, the increased bone formation was not accompanied by a change in the RANKL/OPG ratios.

### Delayed timing of Rs1 expression

The prior results show that Rs1 expression occurs before development of the trabecular bone phenotype at day 5 of postnatal growth. In addition, Rs1 expression after 4 weeks of age (*ColI/Rs1*-late mice, 1*D*) led to no grossly abnormal bone phenotype in adult mice,(11) suggesting a window of sensitivity to Rs1 signaling between postnatal day 5 and 4 weeks of age. We used the tetracycline transactivator system to limit Rs1 expression to narrow the window of maximal osteoblast sensitivity to Rs1 signaling.

To determine if Rs1 expression during the late prenatal to early postnatal period is required to increased trabecular bone formation in adult mice, we suppressed Rs1 expression through birth (*ColI/Rs1*-early mice, 1*E*) by feeding pregnant mice doxycycline-containing chow. Pups and their nursing mothers were removed from doxycycline on the day of delivery to allow Rs1 expression. The pups were followed by serial whole-body BMD scans through the first 9 weeks of postnatal life. The *ColI/Rs1*-early mice developed the trabecular overgrowth phenotype, but to a milder degree than in the *ColI(2.3)*^+^/*Rs1*^+^ mice expressing Rs1 from gestation (see 4*A*), as assessed by whole-body BMD.

**Fig. 4:**
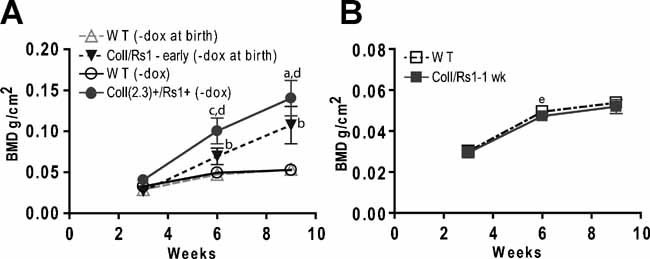
Delayed Rs1 expression leads to a milder bone overgrowth phenotype. (*A*) *ColI/Rs1*-early mice in which Rs1 expression began after birth have intermediate whole-body BMD as compared to *ColI(2.3)*^+^/*Rs1*^+^ mice expressing Rs1 from gestation and similarly treated wild-type littermates. *n* = 4 WT (–dox at birth), 10 WT (–dox), 10 *ColI(2.3)+/Rs1+* (–dox), and 6 *ColI/Rs1*-early (–dox at birth) at each time point. Values are mean ± 1 SD. *a:* *p* < .05 versus *ColI/Rs1*-early; *b:* *p* < .01 versus WT; *c:* *p* < .001 versus *ColI/Rs1*-early; *d:* *p* < .001 versus WT. (*B*) BMD in *ColI/Rs1*–1 week mice and wild-type littermates indicate that the abnormal bone phenotype is not maintained once Rs1 expression is discontinued. *n* = 9 WT and 5 mutant mice per time point. Values are mean ± 1 SD. *e:* *p* < .05 versus WT.

To further define the timing of Rs1 expression necessary to develop and maintain the bone-overgrowth phenotype, we examined *ColI/Rs1*–1 week mice in which Rs1 expression was limited to gestation through the first week of postnatal life (see 1*F*). These mice did not develop the increased trabecular bone as adults or show whole-body BMDs different from wild-type littermate controls (see 4*B*).

These results indicate that maximal Rs1-induced bone formation in an adult mouse is strongly influenced by early Rs1 expression during prenatal gestation and early postnatal development. Furthermore, discontinuing Rs1 expression early in postnatal growth but after the trabecularization has been initiated resulted in normal BMD in adult animals, suggesting that growing bone could recover from the Rs1-induced abnormal bone formation if Rs1 expression was discontinued early.

### Long-term expression of Rs1 in adult mice

The results for the *ColI/Rs1*-early mice and our prior observation that *ColI/Rs1*-late mice (see 1*D*) did not develop the grossly abnormal bone through 30 weeks of age(11) suggested that the ability of Rs1 to induce trabecular bone formation decreased as the mice aged despite detectable Rs1 expression in bone. To identify whether the *ColI/Rs1-*late mice remained free of the bone overgrowth phenotype as the mice aged, we preformed long-term follow-up on these mice through 60 weeks of age.

The *ColI/Rs1*-late mice showed no significant increases in whole-body BMD until 40 weeks of age. Between 40 and 60 weeks of age, all the *ColI/Rs1*-late mice showed increased trabecular bone, but only 50% of the mice developed a whole-body BMD greater than 2 SD above the peak wild-type BMD (see 5*A*). Sites of abnormal bone formation often were adjacent to regions of normal bone and occurred as distinct nodules (see 5*B*). Serum osteocalcin (a marker for bone formation) was increased slightly at 20 and 55 weeks, but serum pyridinoline cross-links (a marker for bone resorption) did not change (see 5*D*). These results suggest that Rs1 activity in adult bone primarily increases bone formation over bone resorption. No significant differences in serum calcium or phosphate levels were detected at either 20 or 55 weeks (data not shown).

**Fig. 5:**
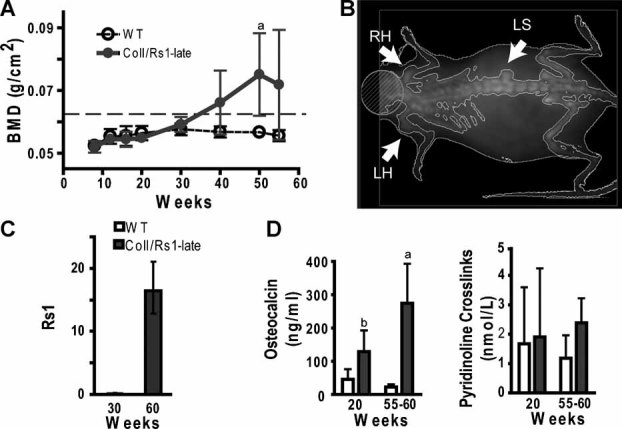
Expression of Rs1 after 4 weeks of age leads to long-term attenuation of the Rs1 phenotype. (*A*) Longitudinal whole-body aBMD showed a significant increase in average BMD as the *ColI/Rs1*-late mice aged. The dotted line indicates 2 SD above the WT BMD at 30 weeks (0.0613 g/cm^2^). *n* ≥ 4 mice for each time point. Values are mean ± 1 SD. *a:* *p* < .05 versus WT. (*B*) DXA image of a representative 60-week-old *ColI/Rs1*-late mouse showing isolated regions of subtle increased bone formation. RH = right humerus; LH = left humerus; LS = lumbar spine. (*C*) Rs1 expression in RNA isolated from whole humeri from representative *ColI/Rs1*-late mice showing low expression at 30 weeks (before the phenotype is evident) and higher expression at 60 weeks (when bone overgrowth is evident). Values represent mean ± 1 SD of technical triplicates. (*D*) Serum analysis of osteocalcin (a marker of bone formation) and pyridinoline cross-links (a marker of bone resorption) in *ColI/Rs1*-late mice. At 20 weeks, *n* = 8 mutants, 6 WT; at 55 to 60 weeks, *n* = 4 mutants, 4 WT. Values are mean ± 1 SD. *a:* *p* < .05 versus WT; *b:* *p* < .01 versus WT.

**Fig. 6:**
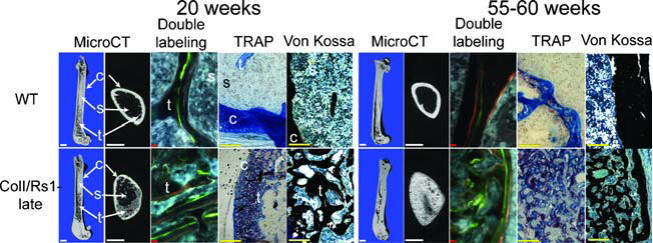
Representative µCT images of femora in longitudinal and transverse mid-diaphysis sections of age- and sex-matched mice show a delayed but gradual increase in trabecular bone and loss of cortical bone in the *ColI/Rs1*-late mice. Scale bar = 1 mm. Double fluorochrome labeling (*middle panels*) with calcein (*green*) and xylenol orange (*orange*) demonstrated disordered bone formation in the areas of increased trabecular bone. Scale bar = 10 µm. TRAP staining (*pink*), indicative of osteoclast-lineage cells, is increased within the regions of expanded trabecular bone in *ColI/Rs1*-late mice. Scale bar = 100 µm. Von Kossa staining shows the increased trabecular bone and loss of normal bone marrow cells (scale bar = 100 µm). c = cortical bone; t = trabecular bone; s = bone marrow space.

µCT analysis on femora from 20-week-old *ColI/Rs1*-late mice showed a subtle expansion of trabecular bone starting at the femoral metaphyses. The increase in trabecular bone was accompanied by a loss of endocortical bone and narrowing of the bone marrow space in the *ColI/Rs1*-late mice (Fig. 6). Double fluorochrome labeling to assess bone mineralization showed an increase in disorganized trabecular bone formation in the 20 and 55-week-old *ColI/Rs1*-late mice. In addition, TRAP staining showed a localized increase in osteoclast-like cells within the regions of disorganized trabecular bone in the *ColI/Rs1*-late mice at both ages despite the normal systemic levels of pyridinoline crosslinks (see 5*D*). Von Kossa staining showed that the regions of disordered trabecular bone in the *ColI/Rs1*-late mice shared similar histologic features with the abnormal bones in the *ColI(2.3)*^+^/*Rs1*^+^ mice expressing Rs1 from gestation, including a decrease in bone marrow cells and an increase in small osteoblast-like cells by morphology.(11)

These results indicate that delaying Rs1 expression until 4 weeks of age leads to long-term attenuation of the Rs1-induced phenotype. However, a subtle increase in bone formation could be detected as early as 20 weeks of age. Although the bone-overgrowth phenotype was clearly evident by 40 to 60 weeks of age, bone formation was significantly slower in the *ColI/Rs1*-late mice than in the *ColI(2.3)*^+^/*Rs1*^+^ mice expressing Rs1 from gestation. In addition, the new trabecular bone appeared to originate from sites of existing trabecular bone, such as at the femoral metaphyses, rather than as de novo formation of trabecular bone at a nontrabecular site, such as at the mid-diaphysis.

### Reversibility of the Rs1-induced trabecular bone formation

The absence of an increased BMD in the *ColI/Rs1*–1 week mice indicated that the Rs1-induced trabecular bone growth might be reversible if Rs1 expression were discontinued before weaning. To determine if established Rs1-induced bone lesions in adult mice also could be reversed and if continued activation of the G_s_ signaling pathway by Rs1 was required to maintain the abnormal increase in trabecular bone, we assessed mice in which Rs1 expression was suppressed after 4 weeks of age (*ColI/Rs1*–4 week mice, see 1*G*). The *ColI/Rs1*–4 week mice developed the abnormal increase in trabecular bone, decrease in cortical bone, and loss of bone marrow space during the first 4 weeks of life. After this bone phenotype was established, we suppressed Rs1 expression by feeding the mice doxycycline-containing chow.

*ColI/Rs1*-4 week femora examined over the next 32 weeks showed a gradual decrease in trabecular bone and a restoration of cortical bone and the bone marrow space (see 7*A, B*). Double fluorochrome labeling showed that the normal linear bone mineralization pattern gradually returned as the trabecular bone disappeared (see 7*C*). TRAP staining also indicated a decrease in osteoclast lineage cells within the bones (see 7*D*). Although the overall shape of the bone did not fully return to normal by 36 weeks of age, the gross deformation originally present in the 4- to 6-week-old mice was significantly reversed. Histology on undecalcified bone sections (see 7*E*) indicated the return of a normal hematopoietic bone marrow space. In addition, we observed a rapid decrease in the number of morphologically small osteoblast-like cells previously identified in the *ColI(2.3)*^+^/*Rs1*^+^ trabecular overgrowth bones.

**Fig. 7:**
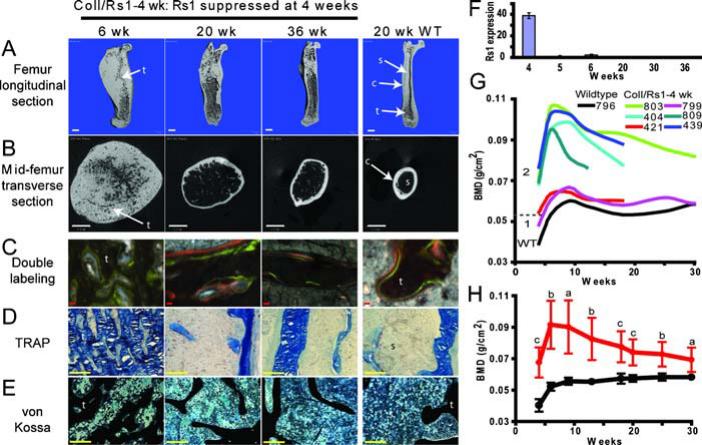
The Rs1-induced trabecular bone phenotype is reversible. (*A*, *B*) Representative longitudinal (*A*) and transverse (*B*) µCT images of femora from *ColI/Rs1*–4 week mice expressing Rs1 from gestation until 4 weeks of age, showing the loss of accumulated trabecular bone and return of the bone marrow space and cortical shell after Rs1 expression has been discontinued. A femur from a 20-week-old WT mouse is shown for comparison. t = trabecular bone; c = cortical bone; s = bone marrow space. Scale bar = 1 mm. (*C*) Fluorescent imaging of double-labeled bone showing the return of linear, orderly bone mineral apposition (*green* = calcein; *orange* = xylenol orange) similar to that seen in WT animals. Scale bar = 10 µm. (*D*) TRAP staining shows a significant decrease in osteoclast lineage cells (*pink*) as the trabecular bone overgrowth resolves. Scale bar = 100 µm. (*E*) von Kossa staining showing the return of normal-appearing trabeculi and bone marrow cells, similar to that seen in WT animals. Scale bar = 100 µm. (*F*) Rs1 expression levels in whole humeri isolated from *ColI/Rs1*–4 week mice shows rapid suppression after doxycycline is started at 4 weeks of age. Representative mice are shown for each timepoint. Values are mean ± 1 SD of technical triplicates. (*G*) Plots of whole-body BMD of six representative *ColI/Rs1*-4 week mice (*colored lines*) and one WT mouse (*black, bottom line*) indicate variation in the degree of bone accumulation at the start of the study at 4 weeks. All mutant mice showed decreases in their BMD after Rs1 expression was discontinued. At 4 weeks, mice were stratified into two groups: BMD < 3 SD above the average WT BMD (0.0521 g/cm^2^, as indicated by the dotted line; WT average = 0.0403 ± 0.0039 g/cm^2^) and BMD > 3 SD above the average WT BMD. Mice in group 2 were selected for pooled analysis in panel *H*. (*H*) Average whole-body BMD for group 2 mice (as determined in panel *G*) showed a continuous decline after Rs1 expression was discontinued. The number of animals meeting the criteria for group 2 at 4, 6, 9, 13, 18, 20, 25, and 30 weeks: WT: 16, 10, 9, 11, 12, 7, 4, and 4; *ColI/Rs1*–4 week: 7, 6, 5, 6, 10, 8, 6, and 5. Values are mean ± 1 SD. *a:* *p* < .05; *b:* *p* < .005; *c:* *p* < .001 versus WT.

BMDs of individual mice at the start of this experiment at 4 weeks of age were variable (see 7*G*), but all BMDs declined or stabilized after Rs1 expression was suppressed. In mice with a starting 4 week BMD of greater than 3 SD above the mean wild-type BMD, whole-body BMDs gradually decreased over 26 weeks, correlating with the reversal of the trabecular bone phenotype observed by µCT imaging (see 7*H*). Serum calcium and phosphate levels at 20 and 36 weeks were similar between wild-type and *ColI/Rs1*–4 week mice (data not shown). Three-week-old *ColI/Rs1*–4 week mice (before starting doxycycline) showed higher levels of serum osteocalcin but not pyridinoline cross-links than age-matched control littermates (359.20 ± 45.91 versus 257.50 ± 50.15 ng/mL, *p* = .01, and 1.42 ± 0.31 versus 1.53 ± 0.42 nmol/L, *p* = .6, respectively; *n* = 5 mutants and *n* = 5 controls). Osteocalcin and pyridinoline cross-link levels were both normal in the 20- to 25-week-old *ColI/Rs1*–4 week mice compared with age-matched control littermates (78.36 ± 44.78 versus 62.91 ± 35.22 ng/mL, *p* = .5, and 1.53 ± 0.63 versus 1.81 ± 0.89 nmol/L, *p* = .6, respectively; *n* = 6 mutants and *n* = 5 controls).

These results indicate that the abnormal trabecular bone formation and loss of cortical bone observed in the *ColI(2.3)*^+^/*Rs1*^+^ mice expressing Rs1 from gestation can be reversed in adult mice by suppressing the expression of Rs1. Although the bone did not return fully to a normal structure at the end of the 36 week experiment, our results demonstrate that adult bone maintains a high degree of plasticity for bone remodeling.

## Discussion

In this study, we applied an engineered receptor approach and mouse model of temporally regulated osteoblast-specific G_s_ activation by the Rs1 RASSL to identify how G_s_ signals regulate skeletal bone formation in mice of different ages. We focused our experiments on the effects of Rs1 expression without ligand activation because Rs1 has a high degree of basal G_s_ signaling activity.(10,11) The double-transgenic “Tet-Off” strategy allowed doxycycline-dependent regulation of Rs1 expression and hence control of the basal G_s_ signaling activity of Rs1. Our studies are especially relevant to the long-term effects of continuous G_s_ signaling by GPCRs in osteoblasts. We found that the Rs1 phenotype developed rapidly between postnatal days 4 and 6, that progressively delayed Rs1 expression resulted in decreased Rs1-induced bone formation, and that the Rs1-induced bone growth and deformities were dramatically reversed when Rs1 expression was suppressed in adult mice.

Our findings with the *ColI/Rs1*-early and *ColI/Rs1*-late mice indicate that osteoblasts from young animals and osteoblasts from older animals both form trabecular bone in response to G_s_ activation by Rs1. However, delaying Rs1 expression leads to quantitatively different amounts of bone formation. Prior results suggested that Rs1 expression may be lower in adult animals,(11) possibly as a result of age-dependent changes in collagen I promoter methylation.(24) Age-dependent decreases in sensitivity to G_s_ signaling or cAMP also may contribute because osteoblast adenylyl cyclase activity decreases with age.(25)

A number of GPCRs are expressed in bone, including the parathyroid hormone/parathyroid hormone–releasing peptide (PTH/PTHrP) receptor,(26) endothelin receptor,(27) prostaglandin E_2_ receptor,(28) adrenaline receptor,(29,30) thyroid-stimulating hormone receptor,(31) and the HTR1b serotonin receptor.(7) Age-dependent activity of these receptors or their ligands may allow other GPCR signaling pathways to modulate the bone-formation effects of Rs1. Activation of the G_i_ signaling pathway by the Ro1 engineered RASSL(22) or other GPCRs(7,32) is associated with decreased trabecular bone formation, suggesting that the G_i_ pathway could dampen the bone-formation effects of G_s_ signaling in older animals. In addition, activation of the G_q_ pathway leads to osteopenia and may desensitize osteoblasts to G_s_ signaling.(33,34) Since the PTH receptor signals through G_s_, G_i_, and G_q_, this suggests that the non-G_s_ pathways balance the G_s_-mediated trabecular bone-formation process. The interactions between the different GPCRs expressed in an osteoblast also may account for the phenotypic variability we observed between individual mice.

Age-dependent increases in osteoclast formation(39) also may contribute to the decreased Rs1-induced trabecular bone formation we observed in adult mice. Although increased markers of osteoclasts are evident in the *ColI(2.3)*^+^/*Rs1*^+^ bone lesions, our results indicate that osteoblast-induced osteoclast formation does not occur during the initial formation of abnormal trabecular bone between days 4 and 5 of postnatal growth. Continued exposure to Rs1 activation eventually may result in increased osteoclastogenesis driven by more mature osteoblasts, similar to what has been reported after PTH treatment of cultured bone marrow stromal cells.(40) As a result, a net increase in osteoclast formation or activity may protect adult bones from the early effects of Rs1.

The bone-formation pattern in the *ColI(2.3)*^+^/*Rs1*^+^ mice expressing Rs1 from gestation resembles the lesions in fibrous dysplasia patients(35) but to a more severe degree. Clinically, fibrous dysplasia lesions are heterogeneous with abnormal and normal trabecular bone, often at distinct anatomic sites.(36) The *ColI/Rs1*-late mice in our study particularly resemble this type of lesion, suggesting that the heterogeneous nature of fibrous dysplasia may be a result of changes in osteoblast sensitivity to G_s_ signaling. This idea that osteoblasts in fibrous dysplasia lesions may have reacquired the characteristics of a perinatal osteoblast correlates with suggestions that fibrous dysplasia may be a stem cell disease.(37) Furthermore, a non-cell autonomous mechanism may contribute to the development of fibrous dysplasia lesions(38) and may similarly modify the osteoblast response to Rs1-mediated G_s_ activation in an age-dependent manner.

Our finding that Rs1-induced trabecular bone formation occurs during a discrete window of days 4 to 5 of early postnatal growth was surprising because the *ColI(2.3)* promoter is active starting before birth,(41,42) and Rs1 expression was detectable before any bone changes were evident on histology. This result suggests that the early developmental processes that establish bone patterning and structure prior to postnatal day 4(43) are not significantly affected by G_s_ signaling. Since the distinct transition at days 4 to 5 of postnatal growth is associated with early weight-bearing activity and the appearance of the secondary spongiosum in mice,(44,45) G_s_ signaling may act at this point to promote trabecular bone expansion. In contrast, G_i_ signaling appears to have roles in neonatal bone formation. Earlier results using the *ColI(2.3)* promoter to express a G_i_ coupled engineered receptor in osteoblasts before birth resulted in embryonic lethality.(22) In addition, *cre*-mediated inactivation of the osteoblast *GNAS* gene encoding the G_s_α subunit resulted in severe neonatal skeletal malformations and decreased trabecular bone.(32) These findings suggest that the roles of G_s_ signaling may shift during early skeletal development from maintenance of the osteoblast niche to driving expansion of trabecular bone.

Our result that the Rs1-induced trabecular bone formation is largely reversible has several important implications. First, bone formation during prepubertal growth can have long-term effects on adult bone, but that bone remodeling may temper these effects. Recent animal studies(46) and clinical studies in children(2) suggest that exercise during childhood can lead to an increase in bone mass that persists with age. The unique nature of the prepubertal skeletal system is further emphasized in that a number of human diseases affecting G protein signaling in the bone have a tendency to present during childhood with long-term sequela in adults.(47–49) However, very early changes in bone formation, as indicated by the *ColI/Rs1*–1 week mice, may not have long-term effects on the skeleton. Second, continuous activation of the G_s_ signaling pathway is required to maintain the increase in trabecular bone induced by Rs1. Clinical studies show that bone formed by recombinant PTH (teriparatide) is lost rapidly unless an antiresorptive medication is used when PTH is discontinued.(50,51) Our results indicate that the changes in bone mass are more likely due to changes in G_s_ pathway activation rather than to the signaling properties of a specific receptor (e.g., PTHR1). Finally, our results show that normal bone has a dramatic ability to repair itself once an insult is discontinued. Since the *ColI(2.3)*^+^/*Rs1*^+^ mice share strong histologic resemblance to polyostotic fibrous dysplasia(35) and the bone lesions in McCune-Albright syndrome,(35) our results suggest that inhibiting the G_s_ signaling pathway may be an effective strategy for treating these patients. Our findings also suggest that bones retain information about their normal shape and structure independent of the capacity for skeletal expansion, which could be used during bone remodeling to restore normal bone structure.

In conclusion, bone formation is a highly plastic process with a complex array of signaling inputs that ultimately determine peak bone mass and normal bone architecture. Understanding how bone tissue can be grown and re-formed may reveal basic properties of bone that will be useful in bone regeneration and repair. Our model provides a dramatic example of how osteoblast-specific hormone signaling can completely reshape bone architecture in a reversible fashion. Insight into how hormone signals can alter morphology will help in the development of new ways to modulate the signaling of endogenous GPCRs to reshape bone in a therapeutic setting.
